# Water-Soluble and Insoluble Polymers, Nanoparticles, Nanocomposites and Hybrids With Ability to Remove Hazardous Inorganic Pollutants in Water

**DOI:** 10.3389/fchem.2018.00320

**Published:** 2018-07-31

**Authors:** Bernabé L. Rivas, Bruno F. Urbano, Julio Sánchez

**Affiliations:** ^1^Departamento de Polímeros, Facultad de Ciencias Químicas, Universidad de Concepción, Concepción, Chile; ^2^Departamento de Ciencias del Ambiente, Facultad de Química y Biología, Universidad de Santiago, Santiago, Chile

**Keywords:** polymers, nanocomposites, membranes, pollutants, water

## Abstract

The polymeric materials have presented a great development in adsorption processes for the treatment of polluted waters. The aim of the current review is to present the recent developments in this field of study by examining research of systems like functional water-soluble polymers and water-soluble polymer-metal complexes coupled to ultrafiltration membranes for decontamination processes in liquid-liquid phase. Noticing that a water-soluble polymer can be turned into insoluble compounds by setting a crosslinking point, connecting the polymer chains leading to polymer resins suitable for solid-liquid extraction processes. Moreover, these crosslinked polymers can be used to develop more complex systems such as (nano)composite and hybrid adsorbents, combining the polymers with inorganic moieties such as metal oxides. This combination results in novel materials that overcome some drawbacks of each separated components and enhance the sorption performance. In addition, new trends in hybrid methods combining of water-soluble polymers, membranes, and electrocatalysis/photocatalysis to remove inorganic pollutants have been discussed in this review.

## Introduction

Several extraction methods are currently applied to remove pollutants from natural water or wastewater. These methods are mainly liquid-liquid extraction, adsorption, precipitation, and others, which are based on the distribution of two phases (Sincero and Sincero, 2002; Stuetz and Stephenson, 2009). Among them, the membrane and adsorption processes are the most developed and mainly use polymeric materials.

A variety of water-soluble polymers are commercially available or can be obtained through synthetic routes. Usually, functional polymers can be obtained by addition polymerization via free radical polymerization (Rivas et al., 2003) or through techniques that provide greater control over the molecular structure such as atom transfer radical polymerization (ATRP) (Matyjaszewski et al., 2000; Li et al., 2005). A functional polymer can be identified as a compound that has functional groups (e.g., carboxylic acid, hydroxyl or amino groups) or as a polymer that performs a specific function (Rivas et al., 2011).

The functional polymers may contain one or more coordination groups, ionizable groups, or groups with a permanent charge in the main chain or side chain. Those polymers that exclusively possess groups that form complexes with metals, such as carboxylic acids, amines, amides, amino acids, alcohols, pyridines, among others, are called polychelatogens (Rivas et al., 2003). The flexibility of these functional groups to interact with the metal ions can be provided by adding spacer groups or increasing the distance of the functional group from the main chain. Similarly, the water-soluble polymers can be a homo- or copolymer, where with an adequate selection of the comonomers, properties such as the solubility, sorption capacity of metal ions, and selectivity can be improved or controlled (Rivas et al., 2011).

Then, the removal of metal or metalloid ions from aqueous sources can be reachedby water-soluble polymers used in combination with ultrafiltration membranes in a technique called liquid-phase polymer-based retention (LPR), which will be described in this review.

**Figure 1:**
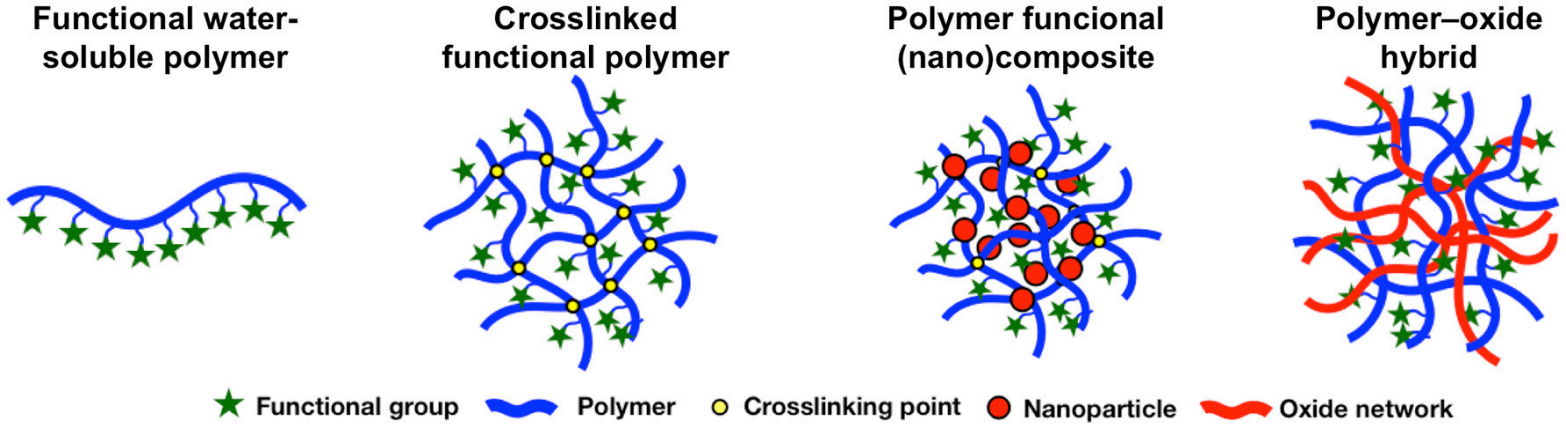
Scheme of the structure of different polymer-based materials for adsorption processes.

The water-soluble polymers can be turned into insoluble compounds by setting a crosslinking point, thus connecting the polymer chains leading to polymer resins (see Figure 1). For decades, organic resins have been demonstrated to be an essential material for the retention of species by ion-exchange. Some advantages are the mechanical and chemical stability, high exchange capacity, and possibility to select the ligand group (Zagorodni, 2007).

Polymer matrices of resins can be of synthetic nature or biopolymers. Similarly, to water-soluble polymers, for their use in the area of heavy metal pollutant removal from aqueous sources, the polymer must possess ionizable groups that allow interaction with an ionic species through electrostatic forces or groups capable of forming a chelate establishing coordination bonds with the metal. In these cases, it is usual to use polymers that have groups such as sulfonic acids, carboxylic acids, ammonium, and amine or chelating agents such as iminodiacetic, N-methyl-D-glucamine, amidoxime, aminophosphonic acids, thiourea, and 2-picolylamine (Da̧Browski et al., 2004; Zagorodni, 2007; Alexandratos, 2009). The incorporation of a moiety of different chemical nature into the polymer matrix, such as one with an inorganic nature, can lead to a composite material that can exhibit novel properties and functions.

A composite material is a material consisting of two phases of different chemical nature (organic, metal, or ceramic), where the phase in greater proportion is the matrix, and the filler corresponds to that in the lowest proportion. When the filler particles are on a nanometer scale, i.e., between 1 and 100 nm in at least one dimension, the resulting material is referred to as a nanocomposite. A hybrid material is understood to be a material in which the organic phase (polymer) and inorganic (e.g., oxide) are mixed at the molecular level and corresponds to a compound with a high homogeneity, where the inorganic phase is often obtained *in-situ* from inorganic precursors through a sol-gel process. A nanocomposite is characterized because the array has scattered discrete structural units (see Figure 1). The IUPAC defines a hybrid material as a material composed of an intimate mixture of inorganic components, organic components, or both types of components (IUPAC, 1997). Meanwhile, a nanocomposite is defined as a composite in which at least one of the phase domains have at least one dimension on the order of nanometers (IUPAC, 1997). Despite these differences, there is an unclear limit of the definitions, and diverse authors use the terms nanocomposite and hybrid interchangeably.

## Nanoparticles, polymer nanocomposites, and hybrids for metal ion sorption

### Nanoparticles

The advances in nanoscience and nanotechnology during the last few decades have developed nanoparticles with different chemical natures, shapes, functionalities, etc. Carbon-based nanoparticles (carbon nanotubes, graphene, and graphene oxide), metal and metal oxide-based nanoparticles (Ag, Au, Fe, TiO_2_, SiO_2_, Fe_2_O_3_, ZrO_2_, ZnO, etc.) and layered silicates (montmorillonite, vermiculite, kaolin, etc.) have exhibited tremendous development due their exceptional properties on the nanoscale (Spitalsky et al., 2010; Rhim et al., 2013; Hu et al., 2014; Liu et al., 2014; Kotal and Bhowmick, 2015). Nanomaterials to be used as adsorbents are interesting because they exhibit a large specific area, high reactivity, fast kinetics and specific affinity to various contaminants (Zhang et al., 2016). For example, carbon nanotubes (CNT) consist of graphene sheets rolled up in a tubular fashion, and according to the synthetic method, single wall carbon nanotubes (SWCNT) or multiwalled carbon nanotubes (MWCNT) can be obtained (Spitalsky et al., 2010). CNT are known for their exceptional mechanical and electric properties as well as their high chemical and thermal stability. To be used as a metal ion sorbent, the graphene layers have to be oxidized to introduce oxygen-containing functional groups that increase the sorption capacity of the carbon material, and in this way CNT have been studied for their ability to adsorb Cu(II), Pb(II), Cd(II), Co(II), Cu(II), Zn (II), Mn(II), Ni(II), and Hg(II) (Li et al., 2003; Chen and Wang, 2006; Stafiej and Pyrzynska, 2007). Additionally, functionalized CNT have been synthesized to enhance the sorption capacity or to provide selectivity to the adsorption, for example amine-based compounds can be attached to the surface of nanotubes for Cu(II) and Pb(II) sorption (Zang et al., 2009; Vuković et al., 2011; Chen et al., 2017; Singha Deb et al., 2017), and a thiol-based compound can be used for Hg(II) sorption due to the high affinity of mercury toward sulfur compounds (Bandaru et al., 2013; Hadavifar et al., 2014; Singha Deb et al., 2017). Meanwhile, other moieties such as iminodiacetic groups (Cui et al., 2012), humic acid (Côa et al., 2017), and cyclodextrins (Hu et al., 2010), among others, can also be used. On the other hand, graphene consists of a single layer of carbon hexagonally arranged, which presents exceptional thermal and electrical conductivity due to the π bonds. These surface characteristics are not favorable to retain metal ions on its surface. However, oxidation into graphene oxide (GO) and the addition of oxygen-containing functional groups to the surface, resulting in a material with a high specific area with the capability for metal ion adsorption. In this way, GO has been used for the adsorption of metal ions such a Cu(II), Cd(II), and Ni(II) (Tan et al., 2015) as well as U(VI) (Wang et al., 2016). Similarly, to the functionalization of CNT, the GO surface has been surface modified with EDTA for Pb(II) sorption (Madadrang et al., 2012) or poly(amidoamine) for Cu(II), (Zn(II), Pb(II), Fe(III), and Cr(III) sorption (Yuan et al., 2013).

The use of metal oxides in adsorption processes is attractive due to their amphoteric character together with their properties at the nanoscale size (large surface area and high activities by the size-quantization effect) (see Table 1). The materials that emerge as attractive nanomaterials for wastewater treatment include iron oxides, aluminum oxides, manganese oxides, titanium oxides, magnesium oxides and cerium oxides, which are the most studied materials (Hua et al., 2012). Iron is an abundant element on the earth, and its facile extraction can produce low-cost iron oxide adsorbents. Goethite (α-FeOOH) (Trivedi et al., 2001; Gerth, 2005; Xu et al., 2006; Brümmer et al., 2013), hematite (α-Fe_2_O_3_) (Jeon et al., 2004; Grover et al., 2012; Ahmed et al., 2013), and maghemite (γ-Fe_2_O_3_) (Ahmadi et al., 2014; Chávez-Guajardo et al., 2015; Madrakian et al., 2015; Rajput et al., 2017) are examples of iron oxides used for metal ion sorption.

**Table 1:** pH_pzc_ for different metal oxides (Fierro, 2005; Kosmulski, 2011).

**Metal oxide**	**pH_pzc_**
Al_2_O_3_	8.0–9.2
TiO_2_ (P25)	6.5–7.0
ZrO_2_	~6.7
SiO_2_ (stober)	< 3.0
Fe_2_O_3_ (maghemite)	6.4–7.5
Fe_2_O_3_ (hematite)	7.0–8.8
FeO(OH) (goethite)	6.5–9.4

Despite the advantageous properties of nanomaterials, their application in large-scale adsorption processes faces several obstacles, especially in flow-through systems such as a fixed bed column or reactive barrier (Zhang et al., 2016). Their inherent tendency to agglomerate, the potential nanoparticle leaching in treated effluent, their separation from purified water and pressure drops are technical issues hard to overcome. An effective solution to these technical issues is to immobilize the nanomaterial onto/into larger particles such as activated carbon, silica particles, and polymers (Zhao et al., 2011).

### Synthesis of nanocomposites and hybrids

The reason for mixing compounds of different chemical nature is the synergy that can be obtained from species with dissimilar properties (e.g., polymer and metal oxide) in only one material. First, the incorporation of an inorganic nanofiller (or carbon-based nanoparticles) into a polymer matrix provides enhancement of the mechanical and thermal properties of the material, or the addition of nanoparticles (e.g., CNT, graphene, etc.) confers electrical conductivity to the polymer (Spitalsky et al., 2010; Kotal and Bhowmick, 2015). For adsorption processes, the incorporation of nanoparticles into the polymer network not only can increase the mechanical strength (or thermal strength) and help to overcome the unfavorable characteristics on the large scale but also add new adsorption sites (Sarkar et al., 2011; Zhao et al., 2011). Furthermore, the synthesis of the polymer can be can tailored to control the porosity, surface chemistry, adsorption active sites, swelling and mechanical properties. Hence, the polymer properties as an adsorbent along with the nanoparticle characteristics result in novel composite materials with superior performance for water treatment.

The synthesis of nanocomposite materials can be obtained through different ways, for example: melt intercalation, solution intercalation, and *in-situ* polymerization (Pavlidou and Papaspyrides, 2008). In melt intercalation, although it is considered environmentally friendly compared to the other synthesis methods, the processing conditions, such as the modification of the filler particles and compatibilizer agents used, play an important role in obtaining a nanocomposite with a proper dispersion of the nanoparticles. However, this process is useful for the synthesis of a polyolefin nanocomposite, but for a polymer possessing functional groups for an adsorption process, it can exhibit degradation and side reactions because of the temperature and the shear forces during the mixing. Additionally, intercalation in solution requires the solubilization of the polymer and the dispersion of the nanoparticles in an appropriate solvent. The agitation of the mixture favors the intercalation of the polymer between the nanoparticles, enhancing the dispersion and distribution of the filler, and then, the polymer nanocomposite is obtained by the removal of the solvent. This strategy seems to be more suitable to obtain a nanocomposite due to the milder conditions compared to melting intercalation; however, this technique (together with melt intercalation) does not allow reticulated polymer to be obtained that provides stabilization to the composite in the sorption process. The technique of *in-situ* polymerization presents several advantages compared to the other strategies because it allows thermoplastics and thermoset polymer nanocomposites to be obtained. In addition, the *in-situ* polymerization provides greater flexibility in the synthesis, thus allowing the modification of nanoparticles on the surface with compounds that favor the dispersion or functionalization of the surface to produce reactions during the polymerization, among others. Additionally, through this technique, it is possible to achieve a higher dispersion of the nanoparticles and allow the formation of a crosslinked polymer hosting the nanoparticles in the polymeric network.

Obtaining a hybrid material, in general, requires the *in-situ* formation of its components (especially the inorganic phase), whereas the organic phase polymer comes from the monomers and the inorganic phase can be obtained from a precursor via a sol-gel process.

The sol-gel process occurs in milder experimental conditions compared to other inorganic materials in a solid state obtained at high temperatures; however, the inorganic solids obtained are usually amorphous, and crystalline phases are distinguishable at the microscopic scale. In this way the sol-gel process emerges as a viable alternative to combine with a polymeric material (Wen and Wilkes, 1996). The sol-gel process is chemically related to a polycondensation, resulting in a three-dimensional network obtained through condensation reactions of small molecules. The process begins with the hydrolysis of an alkoxide of a metal to produce hydroxyl groups, and then, the condensation of the hydroxyl groups occurs to form the three-dimensional network and to generate a residual alkoxide group. Typically, inorganic precursors are the type M(OR)n, where M represents an element of the type Si, Ti, Zr, Al, B, etc. and R is an alkyl group (C_x_H_2x−1_). The structure and morphology of the inorganic network formed depends on several variables, such as the nature of the catalyst, the alkoxide, the acidity, the reactivity of the metal or metalloid, etc. The mild reaction conditions that are required in a sol-gel process together with the broad types of solvents that are compatible allow a polymerization reaction to occur before, during, or after a sol-gel process. In this way, the hybrid can be obtained by i) impregnation of an inorganic precursor in a polymer previously synthesized, (ii) impregnation of monomers (or prepolymers or polymers) in an inorganic compound and subsequent polymerization, and (iii) through a polymerization reaction and simultaneous sol-gel process (Kickelbick, 2007). In the case of the strategies (i) and (ii), the compatibility between the phases is crucial to achieve a mixture at the molecular level, and for this reason, the oxide-polymer interaction should be as similar as possible to the interactions between polymer-polymer and oxide. To this end, the starting materials can be functionalized to facilitate interaction between the phases using reactive and non-reactive coupling agents.

### Metal ion sorption

The polymer matrix of the nanocomposite and hybrid can possess cationic, anionic or chelating groups in the main chain, and depending on them, the nanocomposite will be an anion/cation ion exchanger. Except for chelating groups, these functions are not specific for adsorbing metal ions, and their main role in a nanocomposite is to provide access to the ions by their movement into the polymer through the Donnan exclusion principle (Cumbal and SenGupta, 2005). Furthermore, depending on the nature of the nanoparticles, the compound can exhibit specific sorption toward the metal ions through electrostatic or ligand interactions. The polymer supports often used to prepare these composites are polystyrene crosslinked polymers having fixed cationic or anionic charges (ion exchange resins) with a macroporous structure that are commercially available.

Clays correspond to a type of nanofiller extensively used extensively studied to prepare polymer-clay nanocomposites because of the dramatic changes in properties such as mechanical, barrier and flammability properties of polymers. This compounds are natural ion exchange materials that can be an anion or cation exchanger, such as double layered hydroxide or montmorillonite, respectively, and then, besides their reinforcing character, clays can be a component of a polymeric adsorbent for the removal of Cu(II), Pb(II) and Cd(II) (Say et al., 2006; Kaşgöz et al., 2008; Urbano and Rivas, 2012, 2014) as well as oxyanions such Cr(VI) (Pandey and Mishra, 2011) or As(V) (Urbano et al., 2012a,b). In this case, the addition of clays can enhance not only the sorption properties of the polymer, but also increase the swelling capacity favoring the access of pollutants in the polymer network and the mechanical stability of the sorbent (Zhang et al., 2005; Zheng et al., 2007).

**Figure 2:**
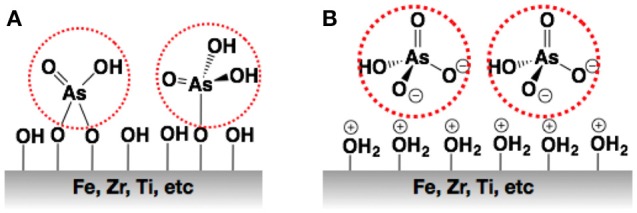
Scheme of the interaction between arsenic oxyanions with the metal oxide surface. **(A)** inner sphere complexes, **(B)** outer sphere complexes.

The polymer-iron oxide nanocomposite is one of the most studied materials for metal ion capture. Iron oxide (nano)particles are considered to be an efficient low-cost adsorbent and geo-chemically important due to their interaction with oxyanions and cations (Zhang et al., 2016). Zhang et al. recently prepared a hydrogel based on hydrous ferric oxide (HFO) nanoparticles imbibed into a porous poly(trans-aconitic acid/2-hydroxyethyl acrylate) via an *in-situ* precipitation method for the sorption of Pb(II), Cu(II), Cd(II), and Ni(II)(Zhang and Li, 2017). In this case, the contribution of HFO nanoparticles was an increase in sorption capacity of the polymer through the formation of inner sphere complexes with the metal cations. Quaternary, ternary and binary ion solutions revealed that the system exhibited more affinity toward Pb(II) and the sorption mechanism was chemisorption (Zhang and Li, 2017). Magnetic separation is one of the promising methods for an environmental purification technique, and hence, the incorporation of magnetic iron oxide leads to adsorbents with the capability to be separated from the mixture using magnetic forces. In this way, several polymer matrices including synthetic and biopolymers have included magnetic iron oxide for the removal of Pb(II), Cd(II), Hg(II), Cu(II), Ni(II), and Co(II) (Zhou et al., 2009; Monier et al., 2010; Fan et al., 2013; Sekhavat Pour and Ghaemy, 2016). Oxyanion contaminants such as arsenate, arsenite, chromate, and vanadate, among others, adsorb onto metal (hydr)oxide surfaces forming stable monodentate or bidentate inner-sphere complexes via ligand exchange with OH^−^or OH_2_ groups (Hristovski and Markovski, 2017). Among the oxyanion pollutants, arsenic (organic and inorganic species) exhibits among the highest toxicity, and its remediation through adsorption and ion exchange techniques has been broadly studied. Particularly, arsenate-ferric (hydr)oxide monodentate complexes exhibits Gibbs energies ranging from −23 to −38 kJ/mol, whereas those of bidentate complexes range from −11 to −55 kJ/mol, and this stable interaction has been exploited for arsenic pollution remediation (Farrell and Chaudhary, 2013). In fact, diverse iron-(hydr)oxide-based adsorbents are commercially available for arsenic sorption (e.g., Bayoxide, GEH-102, NXT-2, etc.) (see Figure 2). The incorporation of iron (hydr)oxide nanoparticles into anion exchange resins bearing quaternary ammonium groups (N^+^R_4_) allows the benefits of both materials to be combined; the nanoparticles provide stable interactions with arsenite and arsenate species, a large specific surface area, and selectivity, while the polymer offers access to the arsenicals into the polymer, ion exchange interaction with arsenate, better hydraulic performance, etc. Hydrated ferric oxide (HFO) nanoparticles were successfully incorporated in cationic and anionic ion exchange resins (DeMarco et al., 2003; Cumbal and SenGupta, 2005; Vatutsina et al., 2007; Iesan et al., 2008), demonstrating that an anionic composite adsorbent offered a substantially higher arsenate removal capacity compared to the cation exchanger (Cumbal and SenGupta, 2005). Li et al. studied the size confinement effect of the host pore structure on the adsorption of As(V) by HFO nanoparticles embedded into anion exchange resins (Li et al., 2016). The main findings were that the diameter of the nanoparticles decreased with the decrease of the average pore size of the reticulated polymer, and this led to an increased sorption capacity and better performance in fixed bed column experiments.

Another oxide nanoparticles recently studied in their ability to retain inorganic pollutant is the zirconium oxide. In this way, ZrO_2_ nanoparticles have been dispersed into a cation exchange resins (sulfonated) and successfully used for Pb(II) sorption, displaying an increased sorption capacity compared to the unloaded resin, and the role of sulfonate groups was enhancing the dispersion of ZrO_2_ nanoparticles together with favoring the diffusion of Pb(II) ions (Zhang et al., 2013a). Similarly, the sorption of Cd(II) ions was enhanced by the incorporation of zirconium phosphate into a strong cation exchanger (Jia et al., 2009). More recently, the capabilities of zirconium (hydr)oxide have been studied toward arsenic sorption (Hristovski et al., 2008; Hang et al., 2012; Cui et al., 2013). It's believed that the interaction of arsenic with zirconium oxide is more stable than that observed for ferric oxide (Pan et al., 2014). In general, the preparation of polymer-zirconium (hydr)oxide nanocomposite consists of loading the inorganic precursor zirconium oxychloride (ZrOCl_2_·8H_2_O) into the polymer, followed by the treatment with alkaline solution to produce the zirconium hydroxide and then heating to form the hydrated zirconium oxide. Polymer–zirconium oxide nanoparticles have been successfully applied in the sorption of As(V), As(III), Cr(VI), and fluoride (Kosmulski, 2002; Mandal et al., 2013; Pan et al., 2013, 2014; Padungthon et al., 2014; Hua et al., 2017). Furthermore, titanium oxide (Borai et al., 2015; Urbano et al., 2015; Mahmoud et al., 2017) and aluminum oxide (Onnby et al., 2012; Praipipat et al., 2017) nanocomposites have been obtained to be used as adsorbents.

Hybrid materials where the organic and inorganic phase are intimately mixed can be obtained for metal ion depollution. A cationic polymer-hydrous zirconium oxide hybrid was recently prepared (Pérez et al., 2016; Toledo et al., 2018). The hybrid exhibited the capability to retain arsenite and arsenate directly, and the sorption to various arsenic species was modulated by changing the inorganic-polymeric composition in the material. Another similar hybrid was prepared using aluminum oxyhydroxide as the inorganic moiety (Pérez et al., 2016). On the other hand, Cr(VI) oxyanions were effectively removed from aqueous solution using a gelatin–silica-based adsorbent material obtained by the sol–gel process (Thakur and Chauhan, 2014). Dong et al. prepared a hybrid sorbet for Cu(II) removal using pyromellitic acid dianhydride (PMDA) and N-[3-(trimethoxysilyl)propyl] ethylene diamine (TMSPEDA) in a sol–gel reaction. The resulting hybrid exhibits anion and cation exchange capabilities (Dong et al., 2011). Likewise, the sorption of Cu(II) was carried out with novel hybrid adsorbentscontaining thiol groups. The hybrid was synthesized through a sol-gel reaction of 3-mercaptopropyl trimethoxysilane in the presence of poly(ethyleneglycol), followed by crosslinking with acetaldehyde (Zhang et al., 2013b). Simultaneous radical polymerization/sol-gel process for obtaining a hybrid based on the copolymer poly[acrylic acid-*co*-3-(trimethoxysilyl) propylmethacrylate]–aluminum oxide was obtained for Ni(II),Cu(II), Cd(II), andPb(II) sorption. The synthesized hybrid had different contents of 3-(trimethoxysilyl) propylmethacrylatecomonomer that in turn acted as a coupling agent, and solely the change of this compound modified the sorption performance of the hybrid (Campos et al., 2015). The sorption of rare earth metals such as La(III), Nd(III), Eu(III), Dy(III), and Lu(III) onto a hybrid material was investigated by Roosen et al. The hybrid was comprised of chiton-silica moieties and further functionalized with ethylenediaminetetraacetic acid (EDTA) as well as diethylenetriaminepentaacetic acid (DTPA) (Roosen et al., 2014). Interestingly, the order of affinity among the metal ions follows the same order corresponding to the stability constants between the lanthanide ions and non-immobilized DTPA (Roosen et al., 2014).

Firstly, we envision that the future research trends in the field of composite adsorbent compounds should focus on the obtaining of materials with a high specific surface to give a high contact area between the adsorbent and the contaminant. Also, to synthesize more stable composites (mechanical and chemically) during the adsorption process, the interaction between the organic-inorganic phases is of greater importance. By setting firm bonds at the organic-inorganic interface, the loss of some of the constituents and the consequent impair performance can be avoided. Secondly, and from a commercial perspective, research on composite adsorbents should be addressed toward their production on a larger scale, competitive in sorption capacity and operating costs. Currently, aluminum- silica, and iron-based compound (as inorganic adsorbent), and ion exchange resins (as organic adsorbents) are widely available in the market for its high sorption capacity and low operational cost. Although, some composite adsorbents compounds have reached their marketing successfully (Lewatit, Ferrix), in general, the complexities of these composite systems increase the costs by making them less attractive to its arrival on the market. In this sense, the development of highly selective adsorbents, novel architectures, high stability, or materials with a significant improvement in the performance compared to existing adsorbents, can find a niche for their development and eventually reach their marketing.

## Water-soluble functional polymers and membranes: LPR technique

The LPR technique easily allows the separation of several species (metal ions, metalloids and organic pollutants), which are bonded to water-soluble polymers. Many applications of water-soluble polymers for the selective separation or concentration of various metal ions from water, have been reported in the literature. The ultrafiltration membrane is the most suitable filter for LPR studies, and a huge amount of data has being reported (Sánchez and Rivas, 2011).

A great advantage of the LPR is the retention of inorganic species in a homogenous medium, avoiding in great part the mass transfer phenomena or diffusion phenomena that are present in a heterogeneous system. For LPR studies, a polymer with a high molecular weight (above 100 kDa) interacts with the anions, and the ultrafiltration membrane retains the new polymer-anion. Normally, poly(ether sulfone) or regenerated cellulose are used as ultrafiltration membranes with a size exclusion molecular weight of 10 kDa, which is much lower than the molecular weight of the polymer. The washing method is similar to a batch-system. A unique polymer:anion solution ratio is put on the cell, and it is washed at constant volume with bi-distillated water at a certain pH. The separation process is evaluated as a percentage of removal [R(%)] versus a filtration factor (Z). The filtration factor is defined as the ratio of the filtrate volume (V_f_) to the cell volume (V_o_) (Sánchez and Rivas, 2011).

### Metal cation removal

The scientific community is interested to find new methods to produce water of quality, using technologies of low cost, easy use, and higher efficiency compared to conventional treatment such as coagulation and flocculation, chemical precipitation, electrochemical treatment, adsorption, and ion-exchange (Asman, 2009; Chen et al., 2009; Doula, 2009; Nanseu-Njiki et al., 2009; Duan et al., 2010; Jiang et al., 2010). The formation of a coordinate bond or ionic bond can be a result of interactions between the functional polymer and metal ions in solution. However, weaker interactions should also be considered (Rivas et al., 2011). The nature of the polymer–metal ion interaction can be classified into two groups. The first group includes the nature of polymer and the second group includes the nature of the aqueous media (Rivas et al., 2011).

The LPR technique with poly(2-acrylamido-2-methylpropanesulfonate sodium) (PAMPS) as sorbent was used for the removal of heavy metal ions from water. The results showed a high retention for various metal ions (Rivas et al., 2001). Despite these good results, the selectivity of the technique can be improved. One alternative for improving these results is the use of copolymer based on AMPS with a nonionic monomer with a higher tendency to coordinate one of several metal ions in solution (Valle et al., 2015). In another study, PAMPS, PVP, a blend of both homopolymers and poly(VP-*co*-AMPS) were studied comparatively as sorbents of Cu(II), Cd(II), and Ni(II), by LPR technique (Valle et al., 2015).

PAMPS is a homopolymer that exhibited the highest retention capacity (around of 60%) of Cu(II), Cd(II), and Ni(II) at pHs 3.2 and 5.7. This behavior can be explained by the electrostatic interaction of metal ions with sulfonate groups located at the external position of the helical structure of PAMPS (Liekens et al., 1997). In addition, the folding of the linear polymers with AMPS groups in its structure avoids the electrostatic repulsion between the sulfonate groups contributing to the accessibility of the metal ions (Fisher et al., 1977).

The (%R) of PAMPS was similar at both pH values (3.2 and 5.7), which indicates that the protons of the media are not interfering on the metal removal. In the pH range studied, the behavior of PAMPS is consistent with the pK_a_ = 1.5 reported for the sulfonic acid groups (Cavus and Gurdag, 2009). At both pH values, the retention profiles decrease slowly as a function of the volume of filtrate. Comparatively PVP and PAMPS exhibited a different behavior toward the metal ions. The PVP contain an electron donor amide functional group that acts as a ligand in the complexation of metal ions (Valle et al., 2015). PVP does not contain ionizable groups and this allows a major folding of the macromolecular chains, which diminished the access of metal ions to PVP compared to PAMPS. It is also probably that amide group of PVP can complex more than one metal ion, while sulfonate groups of PAMPS can complex a single metal ion. The experimental results indicated the retention of Ni(II) was achieved selectively compared to null retention of Cu(II) and Cd(II) at both pH values using PVP at pH 3.2 and 5.7. This behavior suggests a more adaptable macromolecular conformation of PVP to interact with nickel ions, probably because of its smaller ionic radius compared to Cd(II) and Cu(II) (Valle et al., 2015).

The copolymer compared to homopolymers showed different retention behavior toward metal ions. PAMPS presented a high retention capacity of all of the metal ions at both pH values, while PVP showed a selective retention for nickel ions. On the other hand, the retention capacity of copolymer was lower compared to PAMPS. However, the copolymer showed a marked selectivity toward nickel ions and the retention of Cu^2+^ and Cd(II) increased respect to PVP. The mixture of PAMPS and PVP in solution was not efficient for the retention of the studied metal ions (Valle et al., 2015). The preparation of copolymers can be an effective option to modulate the selectivity and maximum retention capacity.

Water-soluble poly(ethyleneimine) [P(EI)] was studied as a sorbent for metal ions in aqueous solutions thought the LPR technique. The LPR technique was performed using a solution containing a mixture of metal cations such as Cu(II), Cd(II), Co(II), Ni(II), Zn(II), Pb(II), and Cr(III). The metal ion removal was studied mainly as a function of pH. It is known that polyamines such as poly(ethyleneimine) P(EI) are suitable macromolecular ligands for the complexation-ultrafiltration of metal ions. In general, the pH has marked influence on the removal of metal ions, showing that when the pH is increased, the retention capacity for metal ions is also increased (Rivas et al., 2012). The results of the polymer-metal ion interaction for P(EI) were as follows: at pH 3, the P(EI) presented selectivity only for Cu(II), reaching 74% retention. At pH 5, the removal of all the metal ions improved, especially in case of Cu(II), which reached 100%. At pH 7, the retention capacities for all of the metal ions were maximized (Cd(II) 99%, Zn^2+^ 98%, Cu(II) 100%, Co(II) 98%, and Ni(II) 100%), with the exception of Pb(II) and Cr(III), which showed no retention. P(EI) exhibited the highest metal retention capacities at higher pH values, where the functional groups could coordinate and remove more easily the metal ions. Cu(II), Cd(II), Co(II), Ni(II), and Zn(II) have similar ionic radii and unfilled *d*-orbitals, which are necessary for bond formation with the amine of the polymers. However, Pb(II) and Cr(III) have more stable electronic structures, preventing complex formation (Rivas et al., 2012). The adequate selection of water-soluble polymers and the previous optimization of media (pH, concentration, and ion strength) can be crucial to achieve selectivity toward metal cations present in aqueous solutions.

### Arsenic removal

Arsenic is present in water mainly as arsenate [As(V)] and arsenite ions [As(III)] which are related to arsenic acid (H_3_AsO_4_) and arsenous acid (H_3_AsO_3_), respectively. The concentrations, forms, and relative ratios of both As(V) and As(III) in water vary significantly depending mainly on changes in the pH and oxidation potential.

Water-soluble polymers containing quaternary ammonium groups have been studied in combination with ultrafiltration membranes as arsenic sorbents. Polymers such as poly[2-(acryloyloxy)ethyl]trimethylammonium chloride [P(ClAETA)] and poly(ar-vinylbenzyl)trimethylammonium chloride[P(ClVBTA)] were prepared and used in LPR technique to remove arsenic. In general, P(ClVBTA) and P(ClAETA) showed higher retention capacities to As(V) species at pH values 8 and 6 compared to 4. At basic pH the divalent ${\text{HAsO}}_{4}^{2-}$ species predominate in solution and can be retained easily by polymers (Sánchez and Rivas, 2011). In addition, different polymer:As(V) molar ratios (31:1, 20:1, 10:1, 6:1, and 3:1) have been studied. In this experiment a constant amount of polymer (7 × 10^−5^ mol) was used at a pH of 8 or 6 (Sánchez and Rivas, 2011). The results for the removal of As(V) by P(ClVBTA) and P(ClAETA) by the washing method presented in Table 2. The results indicated that the retention of As(V) is limited by the polymer concentration when the arsenic concentration. Considering two different orders of magnitude (2 × 10^−4^ and 7 ×10^−5^ mol of polymer) in separate experiments, a typical 20:1 polymer:As molar ratio of was more efficient. It is remarkable that the high efficiency of the polycation is kept constant in the recuperation of As(V) species, even at strong higher arsenic concentrations.

**Table 2:** Effect of the polymer: As(V) molar ratio on the retention of As(V) for the two polymers at a pH of 8 and filtration factor of *Z* = 10.

**Molar ratio polymer:As(V)**	**Mol of polymer**	**Mol of As(V)**	**P(ClVBTA) R(%), pH = 8**	**P(ClAETA) R(%), pH = 8**
(31:1)	7 × 10^−5^	2.25 × 10^−4^	70.0	84.0
(20:1)	7 × 10^−5^	3.45 × 10^−6^	100.0	100.0
(20:1)	2 × 10^−4^	1.00 × 10^−5^	100.0	100.0
(10:1)	7 × 10^−5^	6.90 × 10^−6^	88.0	59.0
(6:1)	7 × 10^−5^	1.12 × 10^−5^	77.0	60.0
(3:1)	7 × 10^−5^	2.25 × 10^−5^	54.0	14.0

#### Polycation-polyanion complexes in aqueous solution for arsenic removal

**Figure 3:**
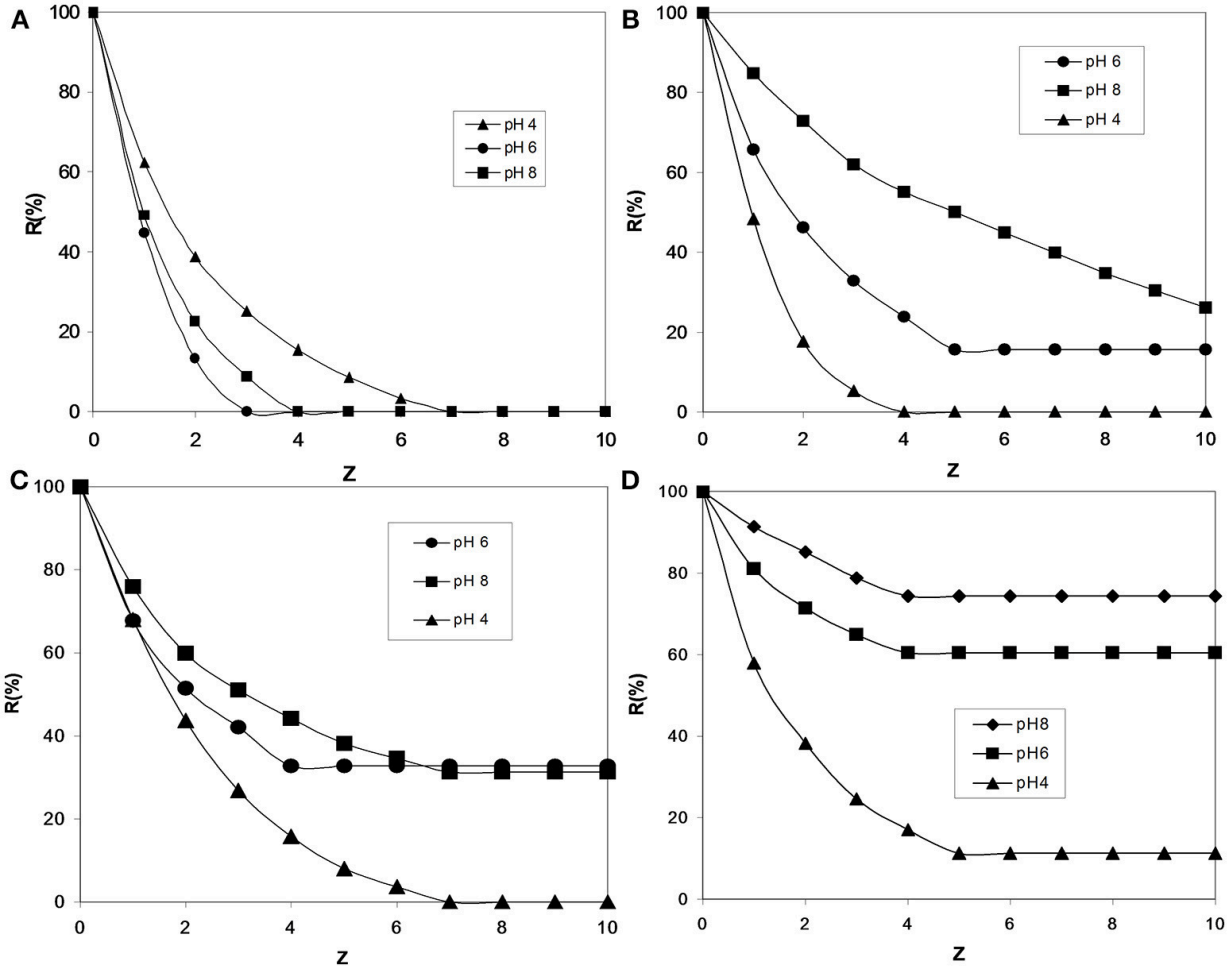
Retention profile of As(V) by poly[(3-methacryloylamine)propyl)trimethyl ammonium chloride-*co*-acrylic acid] [P(ClMPTA-*co*-AA)] at different monomer molar ratios of **(a)** 1:1, **(B)** 1:2, **(C)** 2:1, and **(D)** 4:1. The polymer amount is 0.2 mmol, and the absolute amount of As(V) ions is 0.01 mmol. Adapted from Rivas et al. (2006), Rivas and Aguirre (2007).

Studies about the use of water-soluble copolymers that contain anionic and cationic sites were performed for the retention of As(V) from water. Poly[(3-methacryloylamine) propyl) trimethylammonium chloride-co-acrylic acid] [P(ClMPTA-*co*-AA)]was prepared in various cation:anion molar ratios (1:1, 1:2, 2:1, and 4:1). In this study the retention of arsenic depended mainly of factors such as the pH, polymer concentration and copolymer composition (Rivas et al., 2006). The performance of the polycationic and polyanionic chains in the copolymer was studied toward arsenic retention in function of pH (see Figure 3). When the copolymer prepared at molar ratio of 1:1 was used in LPR, the copolymer loses its ability to bind the As(V) independent of the pH. Based on the literature, a pendant-type polycation can form an equimolar complex with the polyanion. This is probably due to the interaction of the carboxylate groups of the P(AA) with the quaternary ammonium groups of P(ClMPTA) (Rivas et al., 2006). P(AA) is a polymer with proton-donating properties, however in this case its complexation with cationic polyelectrolyes depends of the composition of each unit in the copolymer and also on the pH of the aqueous solution.

In general, P(AA) increased its dissociation in the presence of P(ClMPTA). The dissociation of P(AA) is suppressed at pH of 4 (below the pKa = 5.6). The hydrogen and covalent bonds between cationic and anionic polyelectrolytes hinders the retention of arsenic anions. This is due to the complexation between monomer units, and therefore the retention of arsenate is null.

At a pH of 6, P(AA) is dissociating and forming carboxylate anion and therefore able to produce the complexation. However, the reactivity of the anionic polymer chains covered by cationic polymer chains may be considered higher than the free chain. It is probably due to the changes in the conformation, dissociation and microenvironment in the domain of the polymer chain (Rivas et al., 2006). However, some of the cationic groups are free and available to bind with arsenate of the solution when the copolymer composition is not equimolar. This effect is higher when the ratio polycation:polyanion > 1, where a considerable portion of ammonium groups are free to interact and remove arsenate anions (see Figure 3).

At a pH of 8, the retention of arsenate ions increases for both copolymers without an equimolar ratio. It is observed that when the amount of quaternary ammonium groups increases in the copolymers (4:1), an enhanced retention of arsenic is reached, similar to that of pure P(ClMPTA). The performance of pure P(AA), was found to be negligible in the retention of arsenate anions, independent of the pH (Rivas et al., 2006).

#### Polymer-metal complex for arsenic removal

Lately the development of new water-soluble polymers and its use in combination with ultrafiltration by membranes is considered between the most promissory techniques for enrichment and separation of ions from aqueous solution. On the other hand, the capture of arsenic from aqueous media is important by high toxicity and environmental impact (Sánchez et al., 2015). In addition, for the recuperation of arsenic from contaminated media, new methods on identification and speciation of different form of arsenic have been developed with the aim to improve the levels of sensibility, speed and efficiency on measurements (Thotiyl et al., 2012). In this context our objective was to obtain water-soluble polymers, such as non-cationic system, that is manifested thorough a complex reaction of metallic active site with toxic species to recuperate. For this reason water-soluble complexes of tin (II) salt was prepared in a matrix of poly(acrylic acid), PAA. The tin contents added were 3, 5, 10, and 20 wt %. The tin-metal salt is incorporated to the PAA in order to remove As(III) from an aqueous solution (Rivas and Aguirre, 2007). Sn(II) could coordinate as 2-4 carboxylate groups in structure conventionally bidentate but they are not symmetric. Infrared spectroscopy showed symmetric stretching vibrations ν_sym_(COO^−^) at 1,405 cm^−1^ and stretching asymmetric ν_asym_ (COO^−^) at 1,600 cm^−1^ holding some free carboxylate COO^−^. The bands at 777 and 540 cm^−1^ confirmed the Sn-O bond (Rivas and Aguirre, 2007).

Selectivity studies were carried out to pH 4, 6, and 8 using polymer:As (20:1), (100:1), (200:1), (400:1) and (600:1) molar ratio.

The arsenite retention by P(AA) is negligible, independent of the pH. However, the poly(AA)-Sn presented sorption properties to As(III), confirming that the interaction and removal capacity depended on tin salt concentration, pH of solution, and the polymer-As(III) mole ratio (Rivas and Aguirre, 2007).

At pH 8, As(III) is found anion-dissociated salt in equilibrium with monovalent anions (H_2_AsO${}_{3}^{-}$). Using poly(AA)-Sn (10 wt-% theoretical tin content), 90% of As(III) retention is obtained at Sn-As(III) 20:1 and a poly(AA)-Sn:As(III) 400:1 mole ratio (Rivas and Aguirre, 2007).

**Figure 4:**
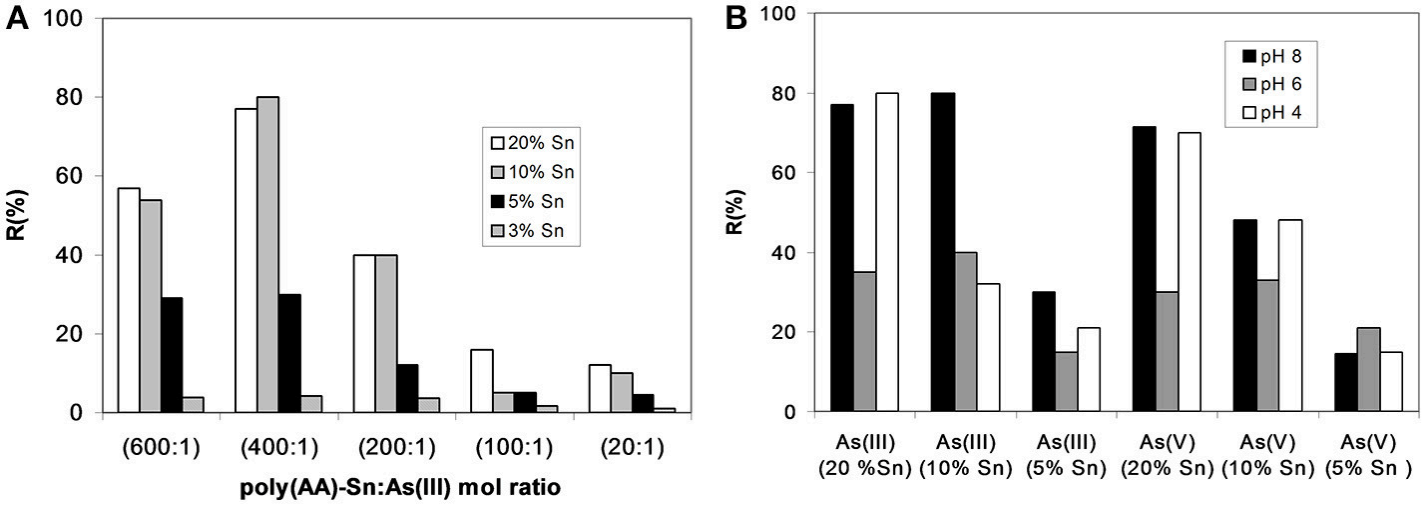
**(A)** Retention values for As(III) at different polymer: As(III) mol ratio and tin content for *Z* = 10 and pH 8. **(B)** Retention values for As(III) and As(V) for *Z* = 10, mol ratio (400:1), at different pH's. Adapted from Rivas et al. (2006), Rivas and Aguirre (2007).

The behavior of different metal-polymer complex (3, 5, 10, and 20 wt% of tin content) is presented in Figure 4. The experiments were carried out with (20:1), (100:1), (200:1), (400:1), and (600:1) poly(AA)-Sn:As(III) mol ratios at pH 8.

When tin content was 3 wt%, the retention degree was negligible. Using higher tin content (5, 10, and 20 wt-%), the retention of As(III) was improved at (600:1) and (400:1) poly(AA)-Sn:As(III) mole ratio. In addition, the higher As(III) retention is double when the tin content is increased (Rivas and Aguirre, 2007).

The higher As(III) removal capacity (up to 90%) indicates that arsenite species are strongly bounded at basic pH, for example when 400:1 at 10 and 20 wt-% tin content was used.

A partial oxidation of As(III) to As(V) is probably due to the higher tin content in the polymer. However, considering the arsenic speciation, majority remains as As(III) with traces of As(V), less than 10 wt-% in the filtrate solution (Rivas and Aguirre, 2007).

The capacity of the polymer to bind arsenite is due to the metal-polymer complexes.

The pH of the media can influence the conformation of the polymer. At higher pH value, PAA is deprotonated and due to electrostatic repulsion of charged carboxylate groups and metal ions are binding with either one or two neighbor groups. At pH 4.8, PAA chains can be contracted and metal ions can be complexed with 2-4 carboxylic acid groups (Rivas and Aguirre, 2007).

Parallel essays by LPR with As(V), on tin metal-polymer complexes, showed a similar profile of retention with As(III). Figure 4 shows the removal of As(III) and As(V) by LPR. The R(%) of arsenic showed that the best performances are found at 10 and 20 wt-% tin content for both arsenic species with a maximum retention at pH 8 and 4 (Rivas and Aguirre, 2007).

Future trends concerning to LPR technique involve the developments of hybrid methods. Those methods include the use of LPR as a pre or post treatment in combined methods such as chemical or electrochemical oxidation/reduction coupled to ultrafiltration using water-soluble polymers (Arar et al., 2014; Sánchez et al., 2015, 2017; Chou et al., 2018). In addition, photocatalysis has been used in combination to LPR to remove improve removal capacities of arsenic (Yuksel et al., 2014; Molinari and Argurio, 2017).

In one study, the chemical reduction was proposed to recover copper from the retentate obtained by LPR technique. Three different polymers (PSS, PAA, and PEI) were studied to explore their effects on the copper removal in LPR and on the copper recovery by chemical reduction under various pH conditions (Chou et al., 2018). The copper removal capacities at pH 3 were different being 60, 75, and 90% for PAA, PSS and PEI respectively. After that, the chemical reduction achieved the complete copper recovery for solution containing PSS. The copper recovery efficiencies were more than 95% for PAA solution with pH values ranging from 3 to 9 at reaction time of 1 h. For PEI, the recovery efficiencies ranged from 20 to 96% and were pH dependent. The results revealed that is possible to remove and concentrate copper from aqueous solution by LPR with different water-soluble polymers and then reduce chemically copper to obtain cuprous oxide in all of the samples (Chou et al., 2018).

In other example, electrochemical oxidation of As(III) to As(V) was performed with metallic electrodes in presence of water-soluble polymers and the ultrafiltration was applied subsequently to remove the total As(V) from aqueous solution (Arar et al., 2014). In the study poly[glycidyl methacrylate N-methyl D-glucamine], P(GMA-NMG), was synthesized and purified by ultrafiltration membranes. It was subsequently used for arsenic removal by coupling electro-oxidation (EO) and LPR processes. At first stage, the complete EO of As(III) to As(V) was performed with a platinum reticulated vitreous carbon-modified electrode and the advancement of the electrolysis was monitored by analytical electrodes. After the oxidation of As(III) to As(V), the LPR wascarried out by washing method using regenerate cellulose membrane and 1 bar of pressure. The results showed that by using P(GMA-NMG) as supporting electrolyte during the complete EO of As(III) to As(V) followed by LPR separation, almost 80% of As(V) was removed at pH 10 (Arar et al., 2014).

The LPR technique was also used in the removal of Cr(VI) ions from aqueous solution using water-soluble poly(diallyldimethylammonium chloride), PDDA, and subsequent electrochemical reduction of Cr(VI) to Cr(III) (Sánchez et al., 2017). The enrichment method in LPR was used to determine the maximum retention capacity of the polymer, and the release of Cr(VI) and regeneration of the polymer were analyzed by sorption-desorption process. Subsequently, the electroanalysis of Cr(VI) was conducted by linear sweep voltammetry and full electrolysis at controlled-potential in acidic media and in the presence of PDDA. The maximum retention capacity of PDDA was 33 mg Cr/g polymer at pH 9. Using the sorption–desorption process, the results indicated that this PDDA can release Cr(VI) and be regenerated. Finally, the electrolysis of Cr(VI) to Cr(III) was performed successfully in the presence of PDDA (Sánchez et al., 2017).

On the other hand, photocatalysis and LPR have been combined to oxidize As(III) to As(V), and then removed by LPR (Yuksel et al., 2014; Molinari and Argurio, 2017). The photocatalytic oxidation of As(III) was performed using TiO_2_ under UV-A light. The As(III) oxidation reached 100% after 30 min of illumination with UV-A light. A water-soluble polymer containing quaternary ammonium groups was used as an extracting reagent in the LPR process. The removal of arsenic by water-soluble polymer was obtained in a 99% yield using a 20:1 polymer:As(V) molar ratio at a pH value of 9. The results demonstrate that the combination of these methods is highly useful for potential applications related to the treatment of wastewater contaminated with As(III) (Yuksel et al., 2014).

## Conclusions

Water soluble and insoluble polymers are versatile materials for water decontamination. Polymer nanocomposites that combine the different properties of inorganic compounds and polymers, can not only enhance the physical properties (for example, mechanical stability), but also, the addition of nanoparticles can provide novel functionalities such ads magnetic characteristics useful to facilitated the separation, or new surfaces of interaction that improve the efficiency and selectivity of the adsorption. Moreover, the polymer matrix allows overcoming operational obstacles that nanoparticles exhibit as adsorbent materials. Similarly, the obtaining of a hybrid composite, where the polymer chains and the oxide network oxide are mixed at the molecular level, are novel materials to be used in adsorption processes.

On the other hand, a non-crosslinked polymer or soluble polymers may also be used for the water decontamination. In this case, the structures of the polymers can be homopolymer or copolymers, and variables such as pH and concentration not only influence the interaction with metal ions but also the polymer chains conformation in solution. A strong polymer-metal interaction will result in a macromolecular aggregate, which can be used in LPR technique.

## Author contributions

JS update the literature and wrote about water soluble polymers and membranes to remove toxic ions. BU update the literature and wrote about nanocomposites. BR organized the whole manuscript.

### Conflict of interest statement

The authors declare that the research was conducted in the absence of any commercial or financial relationships that could be construed as a potential conflict of interest.
